# Advanced electroanalytical chemistry at nanoelectrodes

**DOI:** 10.1039/c7sc00433h

**Published:** 2017-02-17

**Authors:** Yi-Lun Ying, Zhifeng Ding, Dongping Zhan, Yi-Tao Long

**Affiliations:** a School of Chemistry & Molecular Engineering , East China University of Science and Technology , Shanghai , 200237 , P. R. China . Email: ytlong@ecust.edu.cn; b Department of Chemistry , University of Western Ontario , 1151 Richmond Street , London , ON N6A 5B7 , Canada; c State Key Laboratory of Physical Chemistry of Solid Surfaces , Collaborative Innovation Centre of Chemistry for Energy Materials (iChEM) , Department of Chemistry , College of Chemistry and Chemical Engineering , Xiamen University , Xiamen , 361005 , P. R. China

## Abstract

Nanoelectrodes, with dimensions below 100 nm, have the advantages of high sensitivity and high spatial resolution. These electrodes have attracted increasing attention in various fields such as single cell analysis, single-molecule detection, single particle characterization and high-resolution imaging. The rapid growth of novel nanoelectrodes and nanoelectrochemical methods brings enormous new opportunities in the field. In this perspective, we discuss the challenges, advances, and opportunities for nanoelectrode fabrication, real-time characterizations and high-performance electrochemical instrumentation.

## Abstract 2

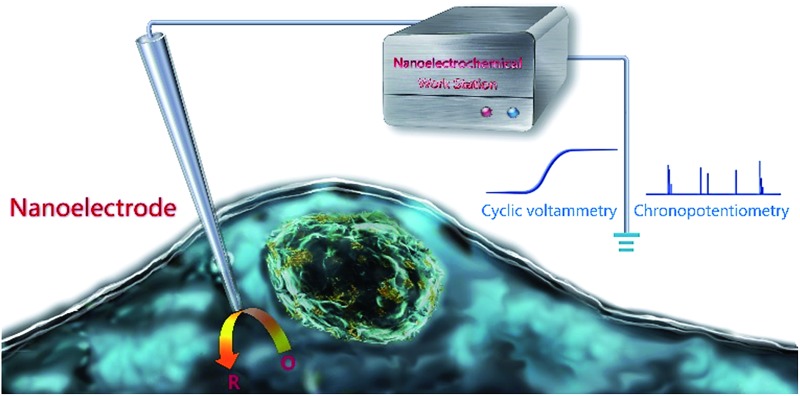
In this perspective, we discuss the challenges, advances and opportunities in electroanalytical chemistry at nanoelectrodes, including nanoelectrode fabrication, real-time characterizations, and high-performance electrochemical instrumentation.

## 1. Introduction

Redox molecules are involved in a variety of charge transfer processes including cellular respiration, cell-to-cell communication, electrocatalysis, and energy conversion and storage. Most of these processes occur at the nanoscale, such as electron shuttles at the mitochondrial membrane^1^ and at the nanointerface of functionalized nanomaterials.^2^ A long-standing requirement in electrochemistry is to develop high spatial resolution and high sensitivity electrochemical techniques to better understand the mechanisms of charge transfer processes at the interface as well as to facilitate related applications. Recently, nanoelectrodes, with dimensions below 100 nm, are regarded as promising tools to study electrochemical processes at the nanoscale.^3^ As the electrode dimensions decrease towards the nanoscale, their geometry becomes smaller than their diffusion lengths. Therefore, mass transport increases and the diffusion layer diminishes. Because radial diffusion becomes dominant, diffusion-limited current is used to describe redox reactions occurring at the nanoelectrode interface.^1^ Moreover, the smaller *RC* (*R* = resistance; *C* = capacitance) time constant (*τ*) of nanoelectrodes ensures their ability to study transient electrochemical reactions.^4^ Because of their advantages, nanoelectrodes have been widely applied in the characterization of single nanoparticles/molecules,^5,6^ the construction of ultrasensitive and non-invasive electrochemical probes for the detection of a single living cell within its natural environment,^7^ and incorporated with scanning electrochemical probe techniques to acquire highly resolved electrochemical imaging.^8,9^

Although nanoelectrode research has achieved excellent progress during recent years, challenges still exist for reliable fabrication and characterization as well as in the development of related instrumentation. For electrochemical analysis at the nanoscale, the surface structure of the nanoelectrodes and their electronic effects must be considered, requiring a very delicate experimental design. If these issues are properly addressed, the understanding of the nanoelectrodes will be greatly improved and their related applications will be bolstered. In this perspective, we discuss the challenges and advances of electroanalytical chemistry at nanoelectrodes. We first highlight typical fabrication methods and their challenges for well-defined nanoelectrodes. Then, we emphasize the characterization methods for nanoelectrodes. The great potential for the development of high bandwidth equipment for nanoelectrodes is also presented. In the last section, we briefly describe the unique applications of nanoelectrodes in single-nanoparticle detection, single-molecule analysis and single-cell probing.

## 2. Fabrication of nanoelectrodes

**Fig. 1:**
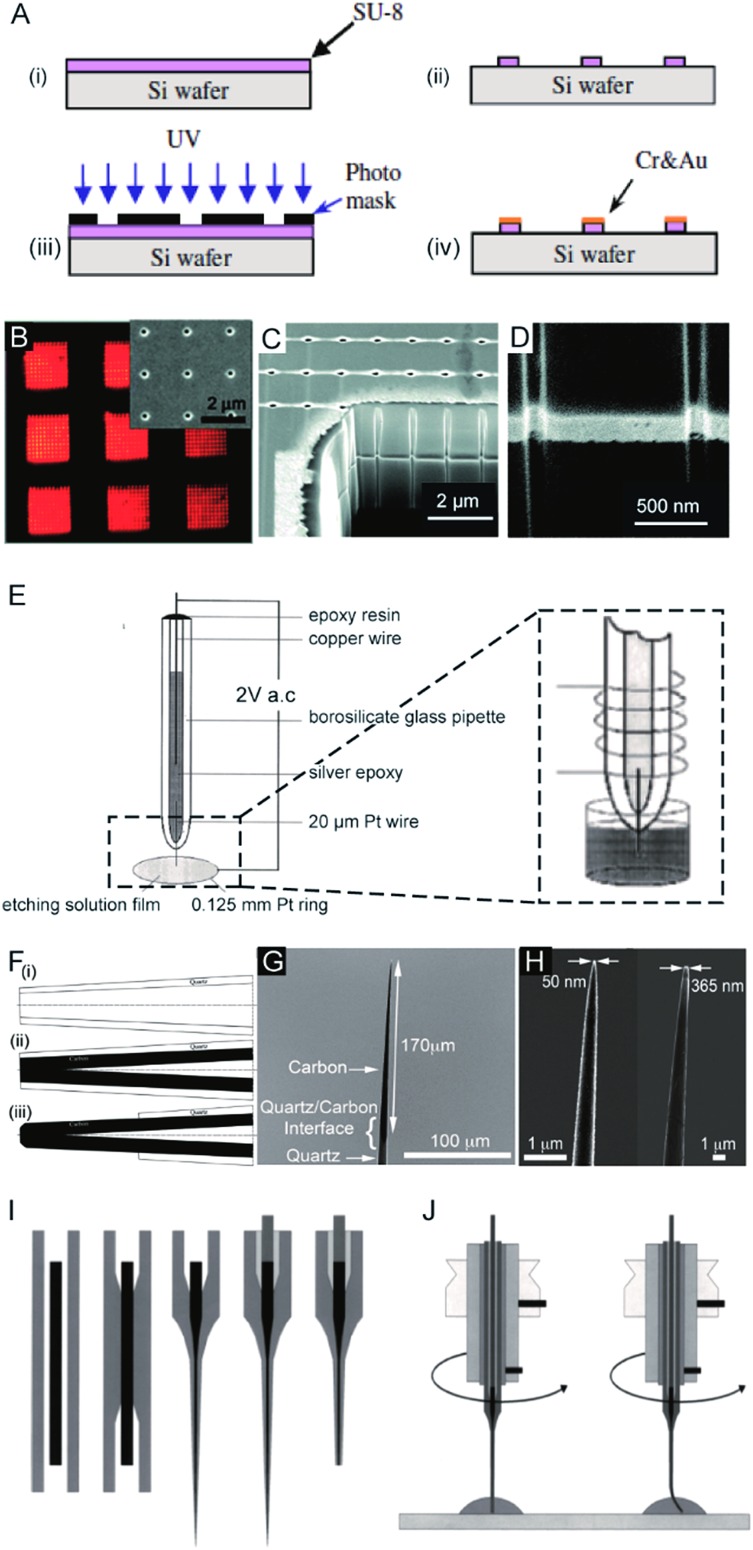
Different fabrication methods for nanoelectrodes. (A) Photolithographic fabrication process for a gold nanoband electrode with (i) spin-coating SU-8 on a silicon wafer, (ii) UV exposure, (iii) SU-8 development, and (iv) Cr and Au deposition onto SU-8.^20^ (B) Reflected light micrograph of nine embedded annular nanoband electrode arrays. Insert: an SEM image of a single pore array with 11 × 11 pores.^22^ (C) The embedded annular nanoband electrode array sample at a 15° tilt with FIB milling.^22^ (D) Magnified view of the arrays at a 52° tilt.^22^ (E) The Pt nanoelectrode produced *via* the electrochemical etching method. A Pt (radius 0.125 mm) ring was covered with a film of etching solution, this film exists stably because of the surface tension. An AC voltage of 2 V was applied between the Pt wire and the Pt ring. The etching procedure was completed when the current dropped to zero.^24^ (F) The fabrication process of carbon nanopipette electrodes by chemical vapour deposition.^38^ (i) The pipette is pulled to the nanoscale; (ii) chemical vapour deposition of carbon; (iii) the quartz/glass at the tip of nanopipette is etched to acquire a carbon tip. (G) An SEM image of the tip profile of a carbon nanopipette electrode.^38^ (H) SEM images of carbon nanopipette electrodes with a tip diameter of 50 nm (right) and 365 nm (left).^38^ (I) Fabrication of a Pt nanoelectrode *via* the laser-assisted wire pulling method. The nanoelectrode fabrication procedure from left to right: the insertion of the Pt-wire into the glass capillary; the cylindrical melting of the quartz glass; the simultaneous pulling of the quartz capillary and Pt-wire; the contacting of the Pt-wire by means of a Cu-wire and a Ag-filled epoxy glue; and the polishing of the obtained nanoelectrode.^45^ (J) The experimental conditions for nanoelectrode polishing from left to right: polishing by rotating the nanoelectrode on a polishing plate in a drop of water or alumina suspension; precession of the fragile electrode tip after pressing the electrode tip onto the polishing plate leads to a conical polishing of the insulating glass sheath.^45^ Adapted with permission from ref. 22 Copyright (2012) American Chemical Society. Adapted with permission from ref. 24 Copyright (2001) American Chemical Society. Adapted with permission from ref. 38 Copyright (2015) American Chemical Society. Adapted with the permission of ref. 20 and 45 from John Wiley and Sons.

The first nanoelectrode was a nanoband electrode reported in 1985.^10^ Thereafter, many types of nanoelectrodes have been reported with various geometries including inlaid disk, hemisphere, sphere, ring, conical, and nanopipette.^11–19^ Four main approaches have been developed to fabricate nanoelectrodes. First, the nanoband electrode (Fig. 1A)^20^ and electrode array were made by using bottom-up manufacturing technologies including photolithography, electron beam lithography (EBL) and focused ion beam (FIB) fabrication,^21^ in which a metal layer was deposited on the top, bottom or sandwiched in-between the substrates. These methods could provide arrays or ensembles of nanoelectrodes.^22^ For example, nanoelectrode arrays on nanochannel arrays with embedded annular nanoband electrodes were fabricated by FIB milling following a layer-by-layer deposition (Fig. 1B–D).

For the second fabrication method, a needle-type nanoelectrode is fabricated by electrochemical etching of thin metal wires down to a cone shape (Fig. 1E)^3,23–25^ or flame-etching of carbon nanofibres for carbon fibre nanoelectrodes.^26–29^ For the third approach, metallic layers are deposited onto the nanoporous polymeric membrane or nanopipette to fabricate the nanoelectrode.^5,15,18,30–34^ For example, a carbon nanoelectrode was fabricated by pyrolysis of a carbon source, such as methane, acetylene, and butane, inside capillaries.^35–37^ Recently, chemical vapour deposition was used to facilitate the deposition of carbon inside the nanopipette (Fig. 1F–H).^38^ Moreover, a nanoelectrode was fabricated by filling a nanocapillary with a Pt ring electrode, which was employed in determining intracellular glucose levels and sphingomyelinase activity.^18^ A nanometric optical fibre was also reported with a metallic coating followed by an insulated sheath of non-conducting polymer films.^39–41^ By using this approach, the dimensions of the nanoelectrode are templated by the pore-formed solid-state nanostructures. These nanoelectrodes can be integrated with electrochemical imaging systems, including scanning ion conductance microscopy (SICM) or optical microscopy techniques. Moreover, this strategy allows for the production of nanoelectrode ensembles. One of the first reported nanoelectrode ensembles was fabricated by an electroless deposition procedure of filling the pores of nanoporous filtration membranes with gold nanowires.^42^

As for the fourth fabrication approach, a laser-assisted wire pulling method was intensively studied to obtain an electrode with a nanoscale radius^43–45^ even down to several nanometers^16,45^ (Fig. 1I). Since this fabrication process takes only a few minutes to seal a commercially available metal wire, it has been widely applied to analyse single entities.^6,46–53^ In detail, a piece of solid metal wire with a diameter ranging between 10 and 100 μm is inserted into borosilicate or quartz glass capillaries and gently heated using the laser puller while applying a vacuum to the two ends of the capillary at the same time. This procedure induces the glass capillary to collapse concentrically, resulting in a tight enclosure of the micrometric wire within the capillary. In the second step, more heat is applied to strongly pull the capillary apart at the two ends while the dimensions of both the glass sheath and the metal wire reduce to the nanoscale. Then, two nearly identical nanoelectrodes are produced at a time. This method has been most commonly used to produce Pt electrodes and to fabricate Au and Ag nanoelectrodes.^44,54^ Usually, a P-2000 Laser-Based Micropipette Puller (Sutter Instrument Co.) is used to achieve an effective pulling process. During the fabrication, the success of this method largely depends on the performance of a specific model of pipette puller. In addition, the experimental environment, including the temperature and humidity, also affects the performance of the pulling procedure. After removing excess glass from the tip by either chemical etching in HF or the electrode polishing procedure, the nanowire is fused in a coplanar and concentric glass insulator, but only the very end of the metal disk is exposed. The polishing procedure is usually operated by rotating the nanoelectrode on a polishing plate in polishing paper or in a drop of water (Fig. 1J).^45^ The final dimensions of the active nanoelectrode are largely determined by the chemical etching or polishing processes, which require very careful operation to avoid unnecessary defects or damage to the nanoelectrode. Even very gentle polishing could change the size and geometry of the nanoelectrode, leading to difficulty in replicating the nanoelectrochemical experiments. For example, a precession of the tip in a circle on the polishing papers was reported to depend on the electrode-to-polishing plate distance.^45^ This conical polishing of the outer glass sheath can be adjusted by changing the angle between the rotation axis of the nanoelectrode and the surface of the polishing plate. Moreover, the following electrochemical measurements should follow precise handling. The unwanted nanometre-scale damage, including electrostatic discharge in the air or electrochemical etching in an electrolyte solution, could induce an inability of current response for the nanoelectrode.^55^ To ensure the sufficient mechanical stability for mechanical polishing, an extended method is developed to insert the pulled nanoelectrode into a second capillary.^16^ Subsequently, this sandwich structure is then pulled again so that the final prepared nanoelectrode is surrounded by a thicker insulating sheath. Moreover, a laser-assisted pipette puller could also be used to fabricate pulled nanopipettes which have been widely used in electrochemical research including for fundamental electrochemical measurements, versatile probes for *in situ* scanning probe microscopy and samplers for ultra-small volumes.^56–60^ Despite tremendous improvements in the fabrication of nanoelectrodes in past decades, challenges still exist to reproducibly, massively produce well-defined nanoelectrodes with controllable geometries and low costs. More importantly, the cleaning of the nanoelectrode surface is difficult because of its small size and extreme fragility in contrast to macroscopic and micrometre-sized electrodes whose surfaces can be reproducibly cleaned by mechanical polishing. A recent study showed the possibility of non-destructively removing an insoluble organic film from nanoelectrode surfaces using an air plasma cleaner.^44^ This method provides an optimized experimental operation for achieving high reproducibility for nanoelectrodes. Therefore, the development of an easy fabrication strategy along with advanced electrochemical experimental protocols is an urgent requirement for further promoting the highly sensitive applications of nanoelectrodes.

## 3. Characterization of nanoelectrodes

The electrochemical performance of nanoelectrodes is extremely sensitive to even small variations in the electrode geometry as well as in the insulating shroud surrounding the electrode. These issues determine the mass transport of the electrode reactant, responding to the proper explanation for the recorded current curve.^61,62^ For example, the steady-state limiting current of a recessed nanoelectrode is smaller than that of a disk nanoelectrode of the same size due to its additional mass-transport resistance.^63^

Usually, by analysing the mass-transport-controlled limiting current (*i*_l_) from the electrochemical method, one can obtain a quick estimate of the size of a nanoelectrode. The value of *i*_l_ is measured from the plateau region of the sigmoidal steady-state voltammogram. The effective electrochemical radius of a hemispherical nanoelectrode could be investigated by using eqn (1), which is based on an assumption about its geometry.^64^

1 *i*_1_ = 2πnFDc*r_app_

where *F* is Faraday’s constant, *n* is the number of electrons transferred in the reaction, *D* is the diffusion coefficient of the reactant (centimetres squared per second), c* is the bulk concentration of the reactant (moles per cubic centimetre), and *r*_app_ is the apparent electrochemical radius (centimetres). Because eqn (1) originates from conventional diffusion theory, it empirically characterizes the apparent electrochemical radius of a nanoelectrode. However, this equation hardly estimates the radius of the nanoelectrode because the nanoelectrode is not a hemisphere. Thus, using eqn (1) would result in an underestimated or overestimated value of *r*. The equation for an inlaid disk electrode and recessed electrode are shown as follows:

2 *i*_1_ = 4nFDc*r_app_

3
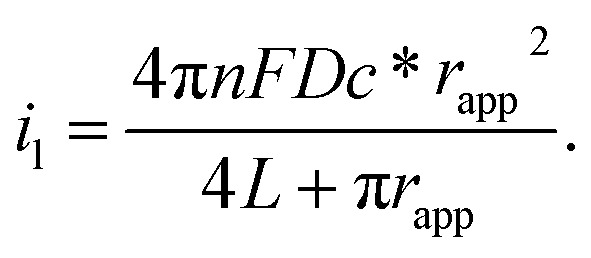



To characterize the truncated conical-shaped glass nanopore electrode, an approximate expression for its diffusion-limited steady-state current is given by eqn (4):^63^
4
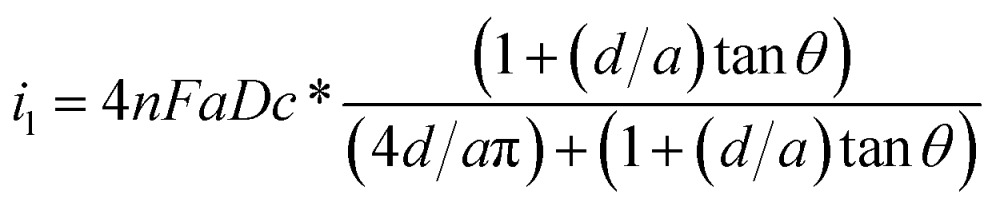
where *a* is the diameter of the pore, *d* is the pore depth and *θ* is the half-cone angle of the pore. It should be noted that the steady-state current formula is inapplicable for the diameter estimation of the nanoelectrode if the electrode morphology is uncertain. Recently, tremendous efforts made to revise the appropriate models for nanoelectrodes, which involve the consideration of a dynamic electrochemical double layer^65,66^ and discussion of the size-dependence of the electron transfer rates.^54^

Moreover, scanning electrochemical microscopy (SECM) has been applied to characterize the shape of the nanoelectrodes based on the use of approach curves.^67,68^ In this experiment, a nanoelectrode is gradually moved closer to a surface in a solution containing electroactive species. At the applied potential, the current acquired from the moving nanoelectrode depends not only on the conductivity of the surface but also on the shape of the approaching nanoelectrode. By comparing the shape of this current–distance curve with those from the theoretical calculations, the shape of the nanoelectrode is revealed.^68,69^ It is important to note that the tip of the nanoelectrode should be within a one or two tip radius distance from the substrate.^70^

**Fig. 2:**
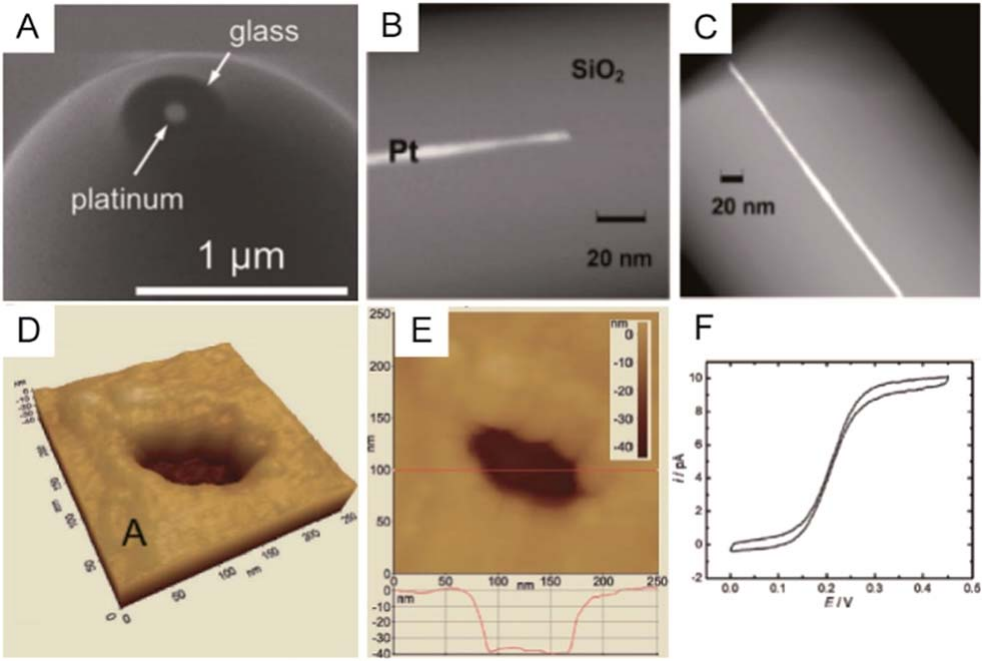
Characterization of nanoelectrodes. (A) SEM image of a ∼110 nm-diameter Pt nanoelectrode;^55^ (B) TEM image of a 1.5 nm radius Pt nanowire sealed in an SiO_2_ capillary;^16^ (C) TEM image of a 3 nm radius Pt nanoelectrode sealed in an SiO_2_ capillary.^16^ Noncontact AFM image of a recessed Pt nanoelectrode in air in (D) 3D and (E) 2D. (F) Steady-state voltammogram of 1.2 mM FcCH_2_OH. The potential sweep rate was *v* = 50 mV s^–1^.^72^ Adapted with permission from ref. 16 Copyright (2009) American Chemical Society. Adapted with permission from ref. 55 Copyright (2013) American Chemical Society. Adapted with permission from ref. 72 Copyright (2012) American Chemical Society.

To clearly visualize the dimensions of the nanoelectrodes, electron microscopy is used to characterize the nanoelectrode. The conventional tungsten-filament SEM images demonstrate the ability to characterize electrodes having a radius larger than ∼50 nm. Field-emission SEM can provide a high spatial resolution below 10 nm (Fig. 2A),^55^ which highly depends on the conductance of the nanoelectrode as well as the skill of the operator. Because the high-energy electrons used in TEM hardly penetrate the thick insulating (∼μm) shroud surrounding the electrode, it is difficult to obtain a clear TEM image of the nanoelectrode.^71^ A previous study shows TEM imaging of a Pt nanoelectrode with overall dimensions smaller than the sub-micrometre scale (Fig. 2B and C).^16^ Although electron microscopy provides the shape and size of a nanoelectrode, there are still obstacles for imaging one tiny nanoelectrode with a radius below 10 nm, much less a massive characterization of capillary-sealed nanoelectrodes. More importantly, it is necessary to uncover the topography of the nanoelectrodes during their operation in solution. Recently, an *in situ* AFM technique was applied to show the recession of an Au nanoelectrode surface into glass after low-temperature annealing.^72^ As illustrated in Fig. 2D and E, a 28 nm radius Pt nanoelectrode has a recess depth of ∼40 nm.^72^ Furthermore, the non-contact AFM image illustrates the cracks in the insulating sheath of nanoelectrode, which may result in solution leakage.^72^ It seems that AFM could be used to characterize the nanoelectrode geometry and surface reactivity in solution. However, if the diameter of the electrode wire is on the order of the AFM tip curvature, it is difficult to distinguish between the contribution of the nanoelectrode and the tip itself. In this case, AFM images are more prone to imaging artefacts rather than a true image of a recessed electrode.

Although a variety of methods have been developed to assess electrode geometries at the nanoscale, nearly none of the presented techniques could provide a comprehensive and real-time picture of the nanoelectrode during its measurements. The question remains of the relationship among the current response of the nanoelectrodes with the real geometry, and the surface absorption and charges for the nanoelectrode during the electrochemical measurements. More effort needs to be directed toward developing novel *in situ* high-resolution characterization methods.

## 4. High performance electrochemical instrumentation

Because the double layer capacitances are proportional to the electrode area, the greatly reduced surface area of nanoelectrodes results in electrochemical cells with small *RC*s. This feature enables the nanoelectrodes to acquire the transient current responses. Note that the effective value of *τ* for the nanoelectrode is dominated by stray capacitances (*C*_stray_) from the electrochemical cell and the electronic board of the current amplifier rather than the electrode capacitance itself.^4^ Therefore, it is important to improve the electronic board of the amplifier and to fabricate the electrochemical cells in a way that minimizes *C*_stray_.

**Fig. 3:**
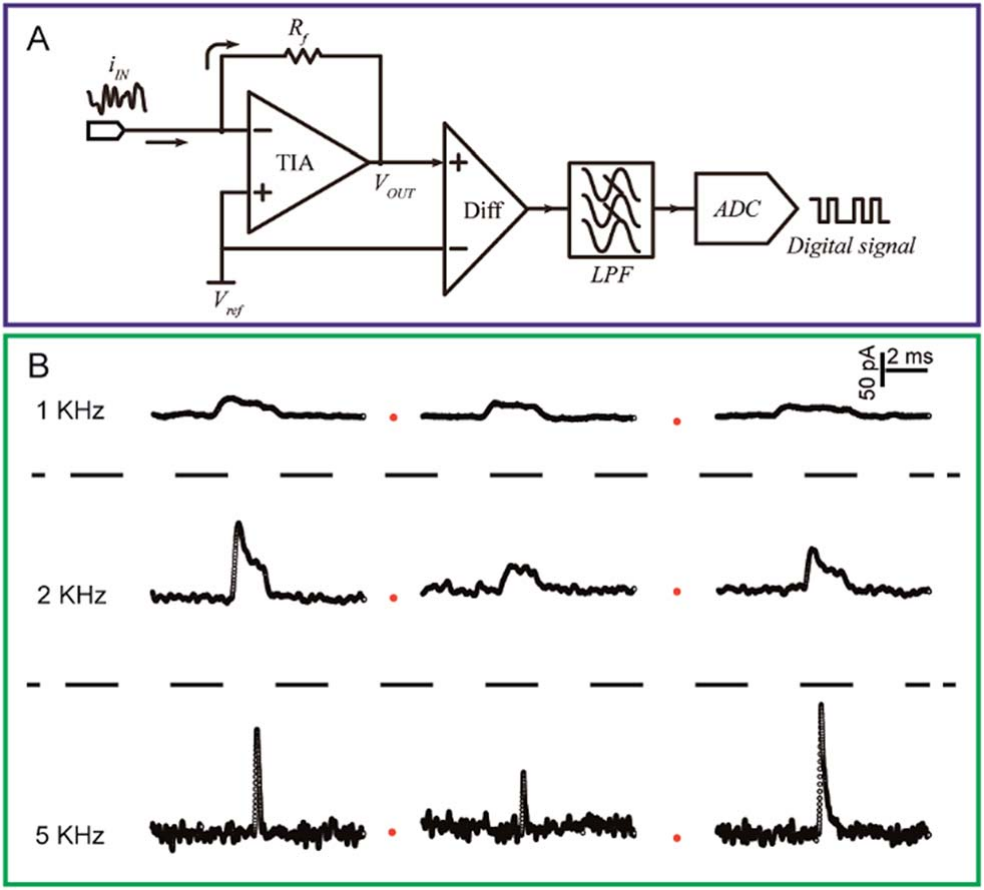
(A) Circuit diagram of a low-current amplifier. During experiments, the bias voltage is set by applying a reference voltage to the non-inverting input of the trans-impedance amplifier (TIA). The current signal is transferred to a voltage signal through the TIA. Then, a unity gain differential amplifier is used to deduct the reference voltage from the signal. To lower the noise, a low-pass filter is always used before the signal is transferred to the digital signal by the analog-to-digital convertor (ADC). (B) Chronoamperometric curves of single 4-mercaptobenzene-1,2-diol@AuNP collisions on a carbon-fibre ultramicroelectrode (UME) recorded at a filter frequency of 1 kHz, 2 kHz and 5 kHz, respectively. The experiments were carried out in a 15 mM phosphate buffer (pH 7.0) containing 5 mM NADH.^74^ Adapted with permission from ref. 74 Copyright (2016) American Chemical Society.

The current response of the nanoelectrodes is typically in the nanoampere to picoampere range, which largely depends on the size of the electrode. Therefore, a high bandwidth system with high gain is essential to amplify the ultra-low current for further recording.^73^ The trans-impedance amplifier (TIA) used for processing the current signal should have an ultra-low input bias current (usually <1 pA) to ensure high-resolution results (Fig. 3A). The input current noise is an important parameter for the amplifier because the noise could be significantly amplified after the current signal is transferred to a voltage signal by the TIA. The noise during the measurement is a result of contributions from the electrode and its associated connections. To evaluate the current fluctuation in nanoelectrode systems, the current–power spectral density, which illustrates the current–power distribution over frequency, could be calculated. Generally, the noise spectrum in nanoelectrode systems is divided into a low-frequency regime and a high frequency regime; whereas, the low-frequency noise includes flicker noise (1/*f*) and thermal noise. The high-frequency noise depends on the capacitance of nanoelectrode, *etc.*

To measure the small and transient current signals accurately, high performance electrochemical instrumentation is desirable with high temporal and current resolution. Otherwise, the recording signal will be deformed by a low bandwidth amplifier, inducing a misleading experimental result. For example, as shown in Fig. 3B, the *i*–*t* response generated from a single functionalized nanoparticle collision was apparently affected by the filter frequency setting of the amplifier.^74^ As the filter frequency decreased from 5 kHz to 1 kHz, the peak current decreased evidently as the duration increased. Recording in the lower bandwidth leads to an obvious change in the shape of the current response.

Normally, current transients are mainly attenuated by the low-pass filter as the filter controls the bandwidth of the amplifier. Blockades with durations shorter than twice the filter’s rise time (2*T*_r_) are prevented from reaching their real maximums. The *T*_r_ of a filter can be estimated by

5 *T*_r_ ≈ 0.3321/*f*_c_

where *f*_c_ is the cut-off frequency of the filter. For the 1 kHz, 2 kHz and 5 kHz low-pass filters, the value of 2*T*_r_ is 0.66, 0.33 and 0.13 ms, respectively. To accurately resolve the current response from the nanoelectrode, the *T*_r_ of the amplifier should be less than the half duration of the signal.

Because the low-pass filter suppresses only the high-frequency noise, the low-frequency noise and noise from the analog–digital converter are still mixed in the recorded current trace. Thus, the existing noise makes it difficult to accurately evaluate each individual current response, especially when studying single entity analysis. Therefore, robust data processes that include locating events as well as evaluating both the duration and the current amplitude should be further developed to provide an accurate analysis of events in nanoelectrode experiments. To achieve this, a true shape of the current response should be well understood. As described by a simple model^75^ for a single nanoparticle collision at the nanoelectrode, the current across the nanoparticle could be regarded as a function of its distance from the electrode surface. Therefore, the true shape for a current spike should be rectangular because the binary current response switches between zero and the limiting current. Considering this model, the recovering of a seriously distorted collision spike could be carried out using full-width-half-maximum (FWHM) methods, the slope of the event or the area of the event, as indicated in previous studies of transient current responses.^76–81^ Given the dynamic redox process at the nanoelectrode,^82^ the true conditions for the single entity analysis are more complicated than this simple model. Therefore, the efficiency of the data evaluation process needs to be continuously explored.

## 5. Applications of nanoelectrodes

Here, we briefly describe nanoelectrode applications in analysing single nanoparticles, studying single molecules and investigating single cells. For a detailed summary of the nanoelectrode applications, we refer the reader to recent excellent reviews including but not limited to ref. 5, 7, 21, 71, 83 and 84.

### 5.1 Single nanoparticles

**Fig. 4:**
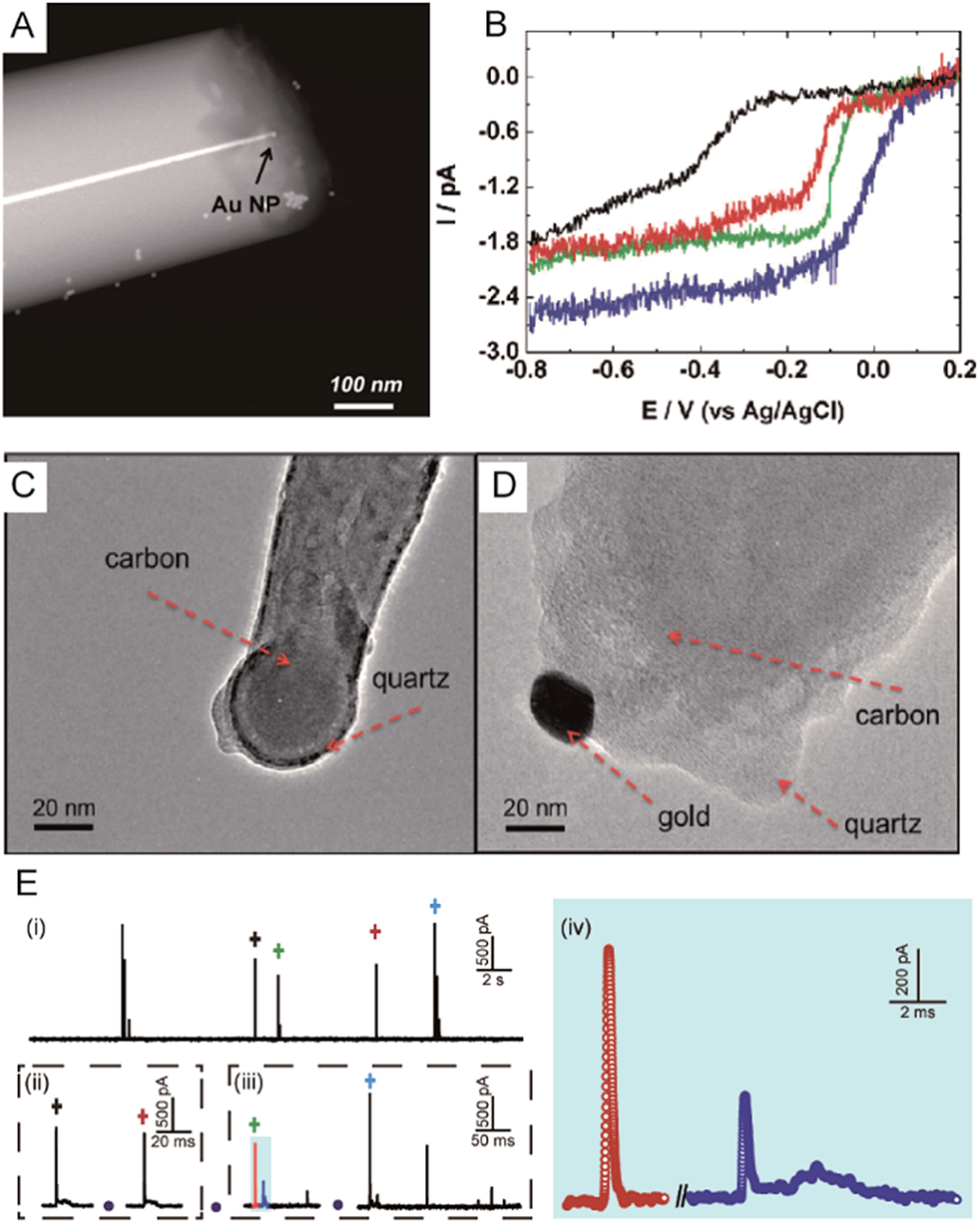
(A) TEM image of a single Au nanoparticle immobilized on a Pt nanoelectrode.^48^ (B) Cyclic voltammetry of a bare 7 nm diameter Pt nanoelectrode (black), a 14 nm Au single nanoparticle immobilized Pt nanoelectrode (SNPE), an 18 nm Au SNPE (green) and a 24 nm Au SNPE (blue) in an oxygen-saturated 0.10 M KOH solution.^48^ TEM images of (C) a carbon nanopipette electrode and (D) a carbon nanopipette electrode with a 20 nm AuNP attached to its tip.^93^ (E) The current transients of 80 nm collisions of individual AgNPs with a nominal diameter on the Au UME (diameter of UME: 12.5 μm) at an applied potential of +0.6 V *vs.* Ag/AgCl QRE.^73^ (i) *i*–*t* transients response; (ii and iii) close-ups of the representative spike clusters shown by crosses with different colours. (iv) Enlarged sections of the red and blue transient in (iii). Adapted with permission from ref. 48 Copyright (2010) American Chemical Society. Reproduced from ref. 93 with permission from the Royal Society of Chemistry. Adapted with permission from ref. 73.

Electrochemical monitoring of single nanoparticle collisions provides detailed information about the nanoparticles including their size, concentration, shapes and diffusion coefficients.^85–87^ The electrochemical measurements of single nanoparticles were performed in both immobilization and nanocollision strategies.^88–91^ For example, single Pt nanoparticles were electrodeposited on carbon nanoelectrodes to readily study the effect of an ultra-high mass-transfer rate for the reduction of oxygen.^92^ As shown in Fig. 4A, a ∼15 nm Au nanoparticle was immobilized on a ∼10 nm Pt nanoelectrode, which was used to study the size-dependent electrocatalytic activity at the single-nanoparticle level.^48^ The results show that the steady-state limiting current increased with the size of the Au nanoparticle ranging from 14, 18 and 24 nm, while the half-wave potential shifted to a higher potential (Fig. 4B). The study of the electrocatalytic activity at a single Au nanoparticle has also been demonstrated on Pt nanoelectrodes with diameters as small as 2 nm.^16^ Recently, a carbon nanoelectrode was used to measure catalytic currents at a single 10 nm gold nanoparticle and at atomic gold clusters (Fig. 4C and D).^93^

The first experiment for a stochastic single-nanoparticle collision was achieved on an UME.^87^ Each collision produced a characteristic current signature that is related to the particle size, the particle residence time, and the particle interactions with the electrode surface. Later, the nanoelectrode was applied in a single nanoparticle collision experiment as a highly sensitive electroanalytical tool. This method has been widely used to study a wide range of nanoparticles from “hard particles”, such as AgNPs,^94^ to “soft particles”, such as transmitter vesicles^95^ (Fig. 4C). Furthermore, the single nanoparticle landing experiments have been carried out using a scanning electrochemical cell microscopy (SECCM) configuration,^96–98^ which ensures a time-resolved detection of the formation of a surface oxide at individual gold nanoparticles.^73^ However, it should be noted that the destructive metal nanoparticle collision towards nanoelectrode/UME undergo complex electrochemical reactions rather than a one-step simple oxidation during the potentiostatic experiment.^82,99–101^ As shown in Fig. 4E, the current responses from the individual 80 nm AgNP collisions on the Au UME show a spike with undulating terrain or multi-spikes. The duration of the first spike of spike cluster in Fig. 4E(ii and iii) is consistent with the time scale of a single encounter, while the whole duration of the event cluster extends to tens of milliseconds. This result is attributed to a series of partially oxidized stages of a AgNP.^82^ Therefore, the complex motion trajectories of the individual nanoparticles should be seriously considered before further applications in the single nanoparticle electrochemical impacts field.

### 5.2 Single molecules

**Fig. 5:**
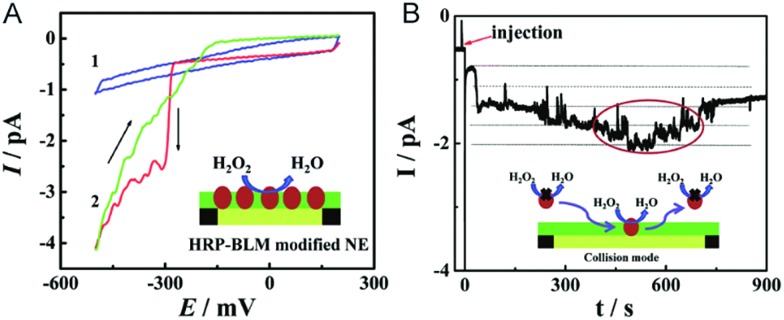
Studying the single HRP with a BLM-Au nanoelectrode.^6^ (A) Cyclic voltammograms of the BLM-Au nanoelectrode (1) and HRP-embedded BLM-Au nanoelectrode (2) in a solution of 20 mM H_2_O_2_ and PBS buffer (pH: 7.4). (B) Chronoamperometric response of HRP collision into the BLM-Au electrode.^6^ Reproduced by permission of ref. 6 from The Royal Society of Chemistry.

The electrochemical detection of a single molecule measures the small electrical current response corresponding to single-molecule redox cycling.^102–110^ It uncovers the internal reaction processes, tracing the intermediate and revealing the dynamics of a single molecule. The first single-molecule electrochemistry study was achieved by trapping single molecules in a 10 nm-thick gap between a conductive substrate and a Pt–Ir nanoelectrode tip.^110^ To analyse the small numbers of a redox-active enzyme, a [NiFe]-hydrogenase film-Au nanoelectrode was fabricated to acquire a distinct catalytic response from less than 50 enzyme molecules.^46^ Recently, a modified Au nanoelectrode demonstrated the efficiency of studying a single horseradish hydroperoxidase (HRP) based on stochastic nanocollision methods.^6^ To biomimic the hydrophobic environment of HRP, a standing bilayer lipid membrane (BLM) was formed spontaneously on the nanoelectrode (Fig. 5A). When HRP collides into the BLM-Au electrode, the HRP-catalysed hydroperoxide reduction generates a staircase current response at the electrode. An estimation of the results reveals that 12 nm Au (Fig. 5B) shows that BLM-modified Au nanoelectrodes provide a good communication pathway between HRPs and the nanoelectrode, which can accommodate 15HRP molecules. Based on the collision events, the turnover number of HRP is calculated to be as high as ∼10^6^ s^–1^.

### 5.3 Single living cells

**Fig. 6:**
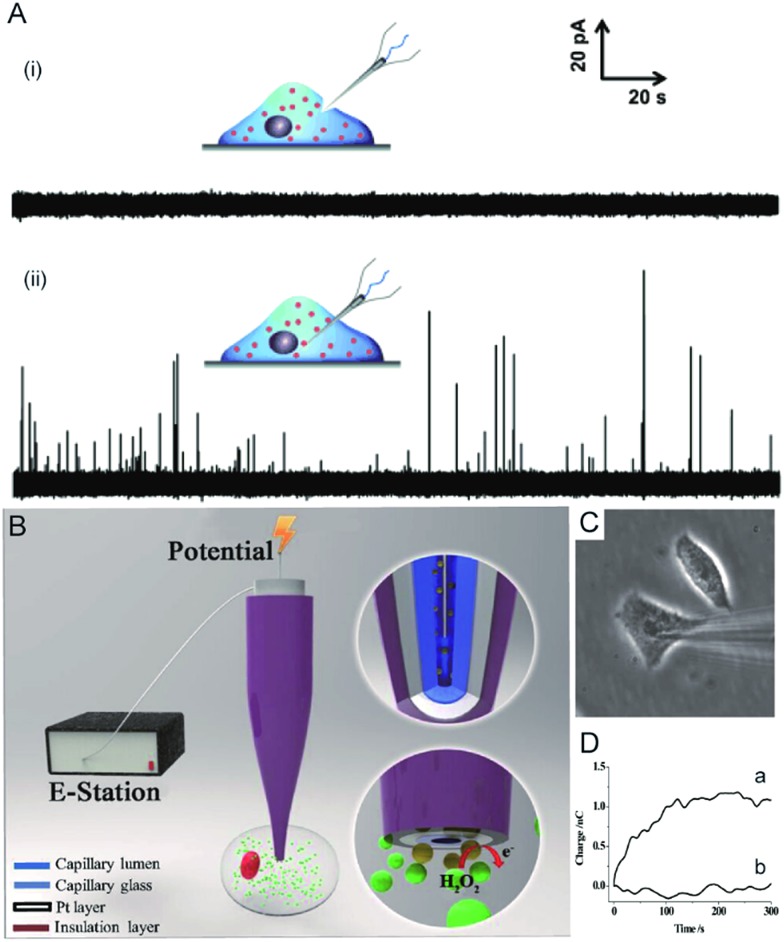
(A) Current responses from a carbon fibre nanotip electrode pushed against a PC12 cell are shown (i) without breaking into the cytoplasm and (ii) inserted into a PC12 cell.^95^ (B) A schematic of the nanoelectrode incorporated nanokit for single cell analysis.^18^ (C) Bright-field image of a nanokit electrode inserted into the cell.^18^ (D) Charges of the nanokit electrode before (a) and after (b) being inserted into the cell.^18^ Reproduced by permission of ref. 18 from Copyright (2016) National Academy of Sciences. Reproduced by permission of ref. 95 from John Wiley and Sons.

Electrochemical techniques are powerful tools for analysing an individual cells’ dynamic redox metabolism because they provide a direct correlation between the signals and electroactive substances in the cells. To achieve the electrochemical detection of a single living cell within its natural environment, the dimensions of the electrodes should be reduced to minimize the mechanical damage to a cell.^7,111–115^ More importantly, the nanoelectrode ensures a high spatial resolution since it can be positioned in different parts of the cell. The results from the nanoelectrodes reflect the amount of reactive oxygen species (ROS) and reactive nitrogen species (RNS) inside the macrophages.^115^ Moreover, nanoelectrodes demonstrate valuable advantages in probing neurotransmitters, most of which are electroactive compounds. A flame-etched carbon fibre nanotip electrode was used to electrochemically measure the total content of electroactive catecholamine in individual nanoscale vesicles in single PC12 cells (Fig. 6A).^95^ The results from the single vesicle collisions with the electrode from inside the cell show that only a fraction of the quantal neurotransmitter content is released during exocytosis. Also, the small dimensions of a carbon fibre nanoelectrode allow for sliding between two neurons membranes, which offers an efficient tool for better understanding of synaptic communication.^116^ Recently, a hollow Pt nanoelectrode was fabricated for filling with components from traditional kits. As a nanoelectrode inserts into a single cell, the components of the kit react with the analyte to generate hydrogen peroxide for the electrochemical measurement. This nanoelectrode-based nanokit was able to determine the intracellular glucose and sphingomyelinase activity (Fig. 6B–D).^18^

## 6. Conclusions and prospective

Recent achievements in the fabrication, characterization and electrochemical instrumentation of nanoelectrodes have dramatically increased their implementation in novel and practical electroanalytical methods.^6,95,115,117–120^ However, more effort is still needed to fabricate high-yield and highly reproducible nanoelectrodes with simple nanofabrication processes. Recent studies show that the thickness and morphology of the surrounding insulator can also have a dramatic impact on the diffusion-limited steady-state current of the nanoelectrodes.^1,121^ Therefore, it is necessary to develop new nanoelectrodes with well-defined structures. Taking the advantage of the defined size of the solid-state nanopore with diameter ranging from sub 2 nm to 50 nm,^122^ one could expect that the nanopore wireless electrode should hold the promising application in nanoscale electrochemistry. Moreover, *in situ* characterization methods that contribute to the understanding of the correlation between theoretical models and the nanostructure of the electrode, including size, geometry and the materials properties for both the electrode and the surrounding insulator, should be established. The complicated electrochemical processes of the nanoelectrodes will be understood through the continued exploration of the integration of the electrochemical platform with other techniques such as surface plasmon resonance (SPR), localised surface plasmon resonance (LSPR), fluorescence microscopy, surface-enhanced Raman scattering (SERS) and mass spectrometry.^123–126^ It should be noted that the limitations of low-bandwidth electrochemical instrumentation hinder the use of nanoelectrodes for the sophisticated study of single molecules and single nanoparticles. In the future, applying and developing high-performance electrochemical instrumentation, as well as related data processing methods, will bolster the further application of nanoelectrodes. For rapidly growing single entity analysis using nanoelectrodes, more research will contribute to a deeper understanding of the structure–activity correlation of each single entity across a variety of chemical/physical transformations.
